# Health-Related Quality of Life and Utility Scores of Posttreatment Patients with Gastric Cancer at Different Pathological Stages: A Cross-Sectional Study

**DOI:** 10.1155/2022/2607829

**Published:** 2022-04-23

**Authors:** Huan Zhang, Chen Sun, Yu Chen, Yan Yuan, Ke Xu, Peipei Lu, Jialin Wang, Nan Zhang

**Affiliations:** ^1^Shandong First Medical University & Shandong Academy of Medical Sciences, Jinan 250021, China; ^2^Centre for Health Management and Policy Research, School of Public Health, Cheeloo College of Medicine, Shandong University, Jinan 250012, China; ^3^Centre for Health Management and Policy Research, School of Public Health, Cheeloo College of Medicine, Shandong University/NHC Key Lab of Health Economics and Policy Research (Shandong University), Jinan 250012, China; ^4^Shandong Cancer Hospital and Institute, Shandong First Medical University and Shandong Academy of Medical Sciences, Jinan, Shandong 250117, China

## Abstract

**Background:**

Health-related quality of life (HRQoL) is a key variable in the evaluation of health economics. We aimed to evaluate the HRQoL and utility scores of patients with gastric cancer and related precancerous lesions by assessing their quality of life using a single standardized health measurement instrument.

**Methods:**

A cross-sectional study was conducted in six counties in Shangdong Province from November 2019 to March 2020. Subjects with precancerous lesions and gastric cancer (cardia and noncardia) were included and surveyed. Patients were divided into four groups: low-grade intraepithelial neoplasia (LGIN), high-grade intraepithelial neoplasia (HGIN), early gastric cancer (EGC), and advanced gastric cancer (AGC). All patients, except those with LGIN, received treatment. The five-level EQ-5D was used to assess HRQoL and generate utility scores using the Chinese-specific tariff published in 2017.

**Results:**

The study included 566 respondents. The average utility was 0.927 for precancerous lesions (LGIN: 0.930; HGIN: 0.926), 0.906 for early gastric cancer (EGC), and 0.756 for advanced gastric cancer (AGC). Visual analogue scale (VAS) means were 76.82 (LGIN: 78.08; HGIN: 74.81), 72.26, and 69.16 for precancerous lesions, EGC, and AGC, respectively. HRQoL was lower in women with AGC than in men (0.612 vs. 0.792, *P* = 0.035). AGC patients were more likely to report problems across all five dimensions than patients in other stages. The proportion of patients reporting pain/discomfort problems was highest across all gastric cancer stages (LGIN, 35.6%; HGIN, 34.4%; EGC, 35.6%; and AGC, 55.7%), followed by anxiety/depression (LGIN, 17.5%; HGIN, 18%; EGC, 22.8%; and AGC, 47.7%).

**Conclusions:**

HRQoL declined as cancer progressed, with the most dramatic decline observed in patients with AGC. A more advanced pathological stage was associated with a greater decrease in health utility. The obtained utilities for different pathological stages of gastric cancer were significant parameters for researchers to perform further cost-utility analysis. Pain/discomfort and anxiety/depression were problems that seriously affected the patients in all groups.

## 1. Introduction

Gastric cancer (GC) negatively impacts human beings, with incidence and mortality ranking fifth and fourth worldwide, respectively, in 2020 [1]. In 2015, it was also the second most common malignancy and the third leading cause of cancer mortality in China [2]. Since the promotion of prevention efforts, including lifestyle improvements and the implementation of screening, and developments in medical science, a stable decline in GC mortality and morbidity has been observed [1]. Thus, treatments are more likely to achieve a better prognosis and health-related quality of life (HRQoL).

HRQoL is a comprehensive subjective indicator system that can estimate physical function, mental health, and social function. Patients' HRQoL deteriorated because of the great suffering caused by the symptoms of gastric cancer such as vomiting and nausea, the treatment intervention process, complications after treatment, and tumor metastasis. Economic evaluation is an analytical method for comparing alternatives in terms of cost and benefit, including cost-minimization analysis (CMA), cost-utility analysis (CUA), cost-effectiveness analysis (CEA), and cost-benefit analysis (CBA) [3]. Health state utility, a significant parameter for generating the quality-adjusted life years (QALYs), plays a crucial role in CUA and CEA [4]. It can be measured using generic and disease-specific methods. Generic measures are preferred because they allow comparisons between different diseases and treatments [5]. There are diverse measurement scales to assess HRQoL, including SF-36, HUI, and EQ-5D. Our study used the five-level EQ-5D (EQ-5D-5L), a generic, preference-based, and multiattribute instrument. Developed by the EuroQol Group, it already has several nationally translated versions and specific utility value systems [6]. It can not only calculate HRQoL but also obtain health state utility. Researchers prefer disease-specific scales because clinical differences are easier to detect. However, these values cannot be applied directly but must be mapped to generic measures [7].

Previous studies have reported that the quality of life differs according to different clinical strategies [8, 9]. Consequently, acquiring patients' HRQoL will aid in assessing and selecting medical technologies. However, most studies have examined the survival time and rate of GC patients after treatment or the difference in HRQoL between various treatments using disease-specific scales [10, 11]. Few investigations have been conducted on the quality of life of patients with precancerous lesions using the EQ-5D-5L to obtain health state utility values. Previous studies have demonstrated the reliability and validity of three-level EQ-5D (EQ-5D-3L) in some cancers, such as breast cancer and esophageal cancer [12, 13]. However, the EQ-5D-5L was developed because of its apparent ceiling effects and sensitivity to certain disease states of EQ-5D-3L [14]. A study comparing the performance between EQ-5D-3L and EQ-5D-5L in breast cancer, colorectal cancer, and lung cancer showed that the ceiling effects decreased in EQ-5D-5L [15]. The purpose of this study was to measure the HRQoL of patients with GC and precancerous lesions and compute their utility scores of them to provide a theoretical foundation for policymakers and economic evaluation.

## 2. Methods

### 2.1. Study Design and Objectives

This cross-sectional study was conducted between November 2019 and March 2020. Six counties covering the eastern, central, and western regions of the Shandong Province were selected as the sample area. Patients diagnosed with gastric cancer (cardia and noncardia) and related precancerous lesions after January 1, 2018, were randomly selected by consulting the hospital information system in specific county-level hospitals. Patients were excluded from the survey if they died before the survey date or refused to participate. All included patients were pathologically diagnosed and divided into four groups: low-grade intraepithelial neoplasia (LGIN), high-grade intraepithelial neoplasia (HGIN), early gastric carcinoma, and advanced gastric carcinoma. All patients except those with low-grade intraepithelial neoplasia completed the primary treatment and progressed to the recovery stage. This study was approved by the Institutional Ethical Review Board of Shandong Cancer Hospital and Institute. All participants provided written informed consent prior to participating in the survey.

### 2.2. Data Collection and Questionnaire

A validated and structured questionnaire was developed to collect relevant patient information, including demographic information (e.g., age, sex, height, weight, and occupation), clinical characteristics (e.g., diagnosis, clinical stage, type of precancerous lesion, and pathological diagnosis code), and HRQoL (measured by EQ-5D-5L). In addition, trained interviewers conducted face-to-face interviews with the patients.

The five-level version of EQ-5D was developed based on the three-level EQ-5D. Previous studies have reported that the measurement properties of EQ-5D-5L are superior to those of EQ-5D-3L, which demonstrated better informativity and convergent and known-group validity [16, 17]. The EQ-5D-5L describes the health status of respondents more accurately and has superior sensitivity [18–20]. It comprises two parts: the EQ-5D descriptive system and EQ visual analogue scale (EQ-VAS) [21]. The descriptive system defines health using five dimensions: mobility (MO), self-care (SC), usual activities (UA), pain/discomfort (PD), and anxiety/depression (AD), each of which has five levels. The five levels correspond to no problems, slight problems, moderate problems, severe problems, and unable to/extreme problems. The combination of these five dimensions and their levels produced 3,125 health states. Each health state can be calculated as a single utility score for all participants through a country-specific standard value set which was estimated based on the native general population. For this study, we employed the Chinese value set, which ranged from -0.391 (state worse than death) to 1.000 (full health) [22]. The EQ-VAS is a 20-cm vertical analogue scale scoring from 0 (representing the worst health you can imagine) to 100 (representing the best health you can imagine). Respondents were required to rate their health on a scale based on their subjective perceptions on the day.

### 2.3. Statistical Analysis

We used descriptive statistics for sociodemographic characteristics. Categorical variables were expressed as percentages and frequencies. Utility scores obtained from the EQ-5D-5L are presented as the mean and 95% confidence intervals (CIs). The chi-square test and Fisher's exact test were used to compare the differences in categorical variables between the various groups. In addition, analysis of variance (ANOVA) and Student *t* test were performed to test the differences in continuous variables. Based on the Chinese value set for the EQ-5D-5L, different domains and different levels had different coefficients (MO = 0.345, SC = 0.253, UA = 0.233, PD = 0.302, AD = 0.258, *L*_1_ = 0, *L*_2_ = 0.191, *L*_3_ = 0.458, *L*_4_ = 0.832, and *L*_5_ = 1), and the utility value calculation formula was as follows: utility = 1 − MO × Ln − SC × Ln − UA × Ln − PD × Ln − AD × Ln (*n* = 1, 2, 3, 4, 5) [23]. The ceiling effect was calculated as the proportion of patients who reported no problems in all dimensions [24]. *P* value <.05 (two-sided) was considered statistically significant. SPSS 25.0 was used to conduct all analyses.

## 3. Results

### 3.1. General Demographic Characteristics of Participants

After exclusion, 566 patients were recruited for this study.  Table 1 presents the primary sociodemographic characteristics of the study population according to disease status, including age, BMI, education, and occupation. Of the 566 eligible patients, 34.3% had LGIN, 21.6% had HGIN, 17.8% had early gastric cancer, and 26.3% had advanced gastric cancer. Most patients were male, comprising 61.9%, 73.0%, 81.2%, and 79.9% of the groups, respectively. Normal and overweight individuals accounted for approximately 80% of all groups, and more than 80% of individuals have an education level of junior high school or less.

### 3.2. EQ-5D-5L Health State Utility Values

The EQ-5D-5L mean, EQ-VAS mean, 95% CI, and ceiling and floor effects are reported in Table 2. The included patients with precancerous lesions had an overall EQ-5D-5L mean utility score of 0.927 (standard deviation [SD] = 0.16, 95% CI: 0.909-0.945), and a VAS score of 76.82. The EQ-5D-5L means of the patients with LGIN and HGIN were 0.926 (SD = 0.01) and 0.930 (SD = 0.01), respectively. The mean EQ-5D-5L index score for EGC was 0.906 (SD = 0.21). The AGC utility score mean was 0.756 (SD = 0.34). As the disease progressed, the EQ-5D-5L utility score significantly decreased from 0.927 to 0.756 as the state of disease progressed (*P* < 0.001), and the mean VAS score decreased from 76.82 to 69.16 (*P* < 0.001).

Almost no difference was detected between the patients with LGIN and HGIN. A slight decrease was observed in patients with EGC, and a sharp drop was observed in AGC patients. The difference in the mean VAS score among the groups was statistically significant (*P* < 0.001). Ceiling effect existed in all groups: 59.3% in the LGIN group, 55.7% in the HGIN group, 56.4% in the EGC group, and 32.2% in the AGC group, and there was a significant difference between groups (*P* < 0.001). Only one patient with AGC reported extreme problems in all five domains.  Table 3 presents the EQ-5D-5L means by gender for all groups. There were no statistically significant differences among the LGIN, HGIN, and EGC patients. However, the EQ-5D-5L mean utility of men with AGC was significantly higher than that of women (*P* = 0.035).

### 3.3. Profile of Five Dimensions of the EQ-5D-5L

**Figure 1:**
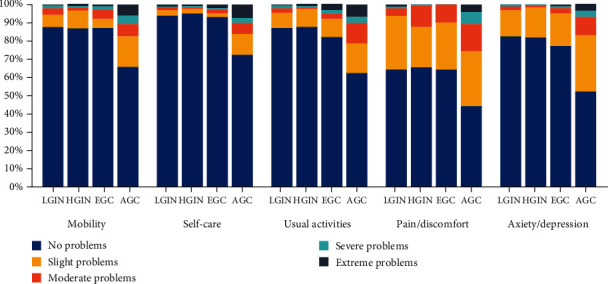
Distribution of EQ-5D-5L responses. Note: LGIN: low-grade intraepithelial neoplasia; HGIN: high-grade intraepithelial neoplasia; EGC: early gastric cancer; and AGC: advanced gastric cancer.

Figure 1 describes the distribution of each domain of EQ-5D-5L by GC stage. Similar profiles were observed in patients with LGIN, HGIN, EGC, and AGC. Pain/discomfort was the dimension with the highest proportion of patients who reported problems at all gastric cancer stages (LGIN, 35.6%; HGIN, 34.4%; EGC, 35.6%; AGC, 55.7%), followed by anxiety/depression (LGIN, 17.5%; HGIN, 18.0%; EGC, 22.8%; AGC, 47.7%). However, self-care had the lowest proportion (LGIN, 6.2%; HGIN, 4.9%; EGC, 6.9%; AGC, 27.5%). In comparison to other groups, a lower proportion of patients with AGC reported having no problems across all five domains.

## 4. Discussion

The current cross-sectional study assessed HRQoL and generated utility values and EQ-5D-5L profiles for patients at different pathological stages of GC. Health utility scores acquired from a single scale can generate QALYs under different health states by combining an individual's survival and quality of life, which are critical for CUA or CEA.

According to our research, patients with precancerous lesions (0.927) and EGC (0.906) had a higher HRQoL than patients with AGC (0.756). The VAS score also reduced steadily as the disease deteriorated from 78.18 in LGIN patients to 61.06 in AGC patients. Our findings confirmed previous research that demonstrated a decline in the quality of life decreased as GC progressed [25, 26]. However, another study found that patients with stage I had the lowest utility value [27]. This could be because most of the gastric cancer patients at stage I in the previous study were treated with surgery, and there was a brief period between surgery and that research. A study conducted in a Portuguese population showed a lower mean utility value in patients with gastric premalignant conditions (0.79) [28]. Different populations, value sets, and larger sample sizes may explain this finding. Xia et al. [26] found that the utility value of patients with high-grade intraepithelial neoplasia was 0.97, which is slightly higher than that found in this study (0.930). The difference existed because they applied the EQ-5D-3L Chinese-specific tariff. Using the EQ-5D-3L Japanese specific value set, Chen et al. [29] reported a 0.797 utility score for AGC patients in the progression-free survival state, which was slightly higher than found in this study. Subgroup analysis suggested that women (0.612) with AGC had lower HRQoL than men (0.792), which is consistent with previous studies [10, 30].

Over half of the patients with LGIN, HGIN, and EGC (59.3% vs. 55.7% vs. 56.4%) and nearly one-third of AGC patients (32.2%), reporting no problems in all five dimensions, described their health as perfect health (also known as the ceiling effect). The ceiling effects were considered distinct in our study because they were all greater than 15% [31], indicating almost no difference among LGIN, HGIN, and EGC patients and a decrease in AGC patients. Compared with patients with LGIN, HGIN, and EGC, patients with AGC frequently present with systemic symptoms, which may explain this phenomenon. According to a systematic review, the EQ-5D scale can more easily identify poor health status or significant changes in health status [32]. Zhou et al. [25] found that the ceiling effect of GC patients was 47.8%, which was higher than that of AGC patients and lower than that of EGC patients in this study, which could be because they included both early and advanced gastric cancer patients. The floor effect was observed in patients with AGC but not in LGIN, HGIN, and EGC patients; one patient (0.7%) stated that he was in the worst health state.

Our study found that AGC patients were more likely to report problems across all five dimensions than LGIN, HGIN, and EGC patients. There was little difference in the proportion of patients with LGIN, HGIN, or EGC reporting problems. Patients with AGC had a lower quality of life and worse life experiences than those with LGIN, HGIN, and EGC. The dimension where patients were most likely to report problems was pain/discomfort in all groups in this study, followed by anxiety/depression. This finding was supported by data showing that pain/discomfort was also the domain with the highest proportion of reporting problems, whereas anxiety/depression was the second [26, 27]. Approximately, 40% of cancer survivors experience pain due to cancer treatment and complications [33]. Pain is a common symptom that significantly influences the HRQoL of patients and is difficult to manage through advancements in treatment technology. A previous study suggested that pain is a prognostic factor for the survival of patients with GC [34]. Anxiety and depression, caused by fear of diagnosis, antitumor treatment, and recurrence, also lead to a poor quality of life in cancer patients. Cha et al. [35] conducted a structural equation model and path analysis, which found that depression had the greatest direct negative impact on quality of life and that distress and insomnia indirectly exerted an adverse effect on the quality of life through depression. Approximately 20.75% of GC patients experienced anxiety and/or depression before laparoscopic surgery, and 44% of disease-free stomach cancer survivors experienced depression after surgery for more than one year [36, 37]. Depression also leads to a worse prognosis in GC patients. Huang et al. [38] reported that patients with GC without depression had significantly longer median survival times than those with depression. Bamonti et al. [39] reported that a quarter of cancer survivors experienced major depression and that pain increased their risk of developing depression in cancer survivors. Effective pain management and psychotherapy interventions are urgently needed to help GC patients live a more comfortable life and improve their quality of life. According to our study, the self-care dimension had the highest proportion of reporting no problems across all dimensions, although the proportion of patients with AGC who reported no problems was lower than that of LGIN, HGIN, and EGC. Abdel-Rahman [40] demonstrated that the dimension where AGC patients were most likely to report no problems was self-care (74.5%), which was similar to our finding (72.5%).

This study had two strengths. First, the Chinese-specific EQ-5D-5L tariff published in 2017 was used, and all patients were pathologically diagnosed. The health state utilities obtained were more accurate. Second, we examined all stages associated with an increased risk of developing gastric cancer, including precancerous lesions, particularly LGIN, a stage that has received little attention in the literature. Thus, we can provide more coherent parameters for further studies on CEA and CUA.

The current study had several limitations. First, the ceiling effect in each group was relatively high, which indicated that many respondents chose an extreme health state (perfect health), and the HRQoL of patients was not fully detected. However, several studies demonstrated that the sensitivity of the EQ-5D-5L scale was confounded, limited its capability to some extent [41–43]. It has been shown that utility values vary widely between different instruments and that generic measures are better used together with disease-specific measures such as the European Organization for Research and Treatment of Cancer Quality of Life Utility Measure-Core 10 dimensions, a utility-based cancer-specific instrument [44, 45]. Second, our results may be affected by demographic characteristics, which were not comparable between groups. In this study, all respondents were from the same province, which may have affected the extrapolation of our findings to some degree. More emphasis should be placed on multicenter research in the future. Finally, we were unable determine whether the differences between the groups are clinically significant because of the lack of evidence against the Chinese-specific EQ-5D-5L tariff. In conclusion, the present study demonstrates that the severity of gastric cancer is associated with a patient's quality of life. The HRQoL of AGC patients was severely impaired compared with that of LGIN, HGIN, and EGC patients. Women with AGC had a lower HRQoL than men. Implementing early diagnosis and treatment of cancer is necessary for patients with GC to maintain a better quality of life. In future clinical practice, more emphasis should be placed on pain management and psychological counseling of gastric cancer patients. Furthermore, we also obtained the health utility index and provided some parameters for health economic evaluations by incorporating them into Markov models.

## Figures and Tables

**Table 1:** Social demographic characteristics of study population.

	LGIN (*n* = 194)	HGIN (*n* = 122)	EGC (*n* = 101)	AGC (*n* = 149)	Total (*n* = 566)
Gender (%)					
Male	61.9	73.0	81.2	79.9	72.4
Female	38.1	27.0	18.8	20.1	27.6
Age (years, %)					
≤55	25.8	18.0	11.9	15.4	18.9
56-64	38.7	39.3	35.6	36.9	37.8
≥65	35.6	42.6	52.5	47.7	43.3
BMI (kg/m^2^, %)					
Underweight	4.1	13.1	9.9	32.9	14.7
Normal	44.8	45.1	65.3	51.0	50.2
Overweight	40.2	33.6	17.8	11.4	27.2
Obese	10.8	8.2	6.9	4.7	8.0
Education level (%)					
Primary school and below	45.4	41.8	47.5	43.0	44.3
Junior high school	39.7	47.5	37.6	34.9	39.8
Senior high school	14.9	9.8	13.9	17.4	14.3
College and above	0.0	0.8	1.0	4.7	1.6
Marital status (%)					
Unmarried	0.5	3.3	1.0	0.7	1.2
Married	92.8	89.3	94.1	93.3	92.4
Divorced	0.0	1.6	0	0.7	0.5
Widowed	6.7	5.7	5.0	5.4	5.8
Occupation (%)					
Peasant	90.2	91.0	87.1	75.2	85.9
Others	9.8	9.0	12.9	24.8	14.1
Annual personal income (yuan, %)					
<5000	53.1	63.9	56.4	53.7	56.2
5000-10000	25.3	16.4	23.8	17.4	21.0
>10000	21.6	19.7	19.8	28.9	22.8

Note: LGIN: low-grade intraepithelial neoplasia; HGIN: high-grade intraepithelial neoplasia; EGC: early gastric cancer; AGC: advanced gastric cancer; and BMI: body mass index was classified into 4 categories, underweight (<18.5 kg/m^2^), normal (≥18.5 and ≤23.9 kg/m^2^), overweight (≥24 and<28 kg/m^2^), and obese (≥28 kg/m^2^).

**Table 2:** EQ-5D-5L utility and VAS scores of four groups.

	EQ-5D-5L mean (95% CI)	EQ-VAS mean (95% CI)	%ceiling	%floor
LGIN	0.926 (0.901,0.950)	78.08 (75.77,80.40)	59.3	0
HGIN	0.930 (0.904,0.955)	74.81 (71.87,77.75)	55.7	0
EGC	0.906 (0.865,0.946)	72.26 (68.80,75.71)	56.4	0
AGC	0.756 (0.701,0.810)	69.16 (65.78,72.54)	32.2	0.7
P value	<0.001^a^	<0.001^a^	<0.001^b^	0.657^c^

Note: LGIN: low-grade intraepithelial neoplasia; HGIN: high-grade intraepithelial neoplasia; EGC: early gastric cancer; AGC: advanced gastric cancer; CI: confidence interval; ^a^ANOVA test was used; ^b^Chi-square test was used; ^c^Fisher's exact test was used.

**Table 3:** EQ-5D-5L index score according to gender in four groups.

	Male mean (95% CI)	Female mean (95% CI)	*P*
LGIN	0.914 (0.876,0.951)	0.945 (0.925,0.966)	0.146∗
HGIN	0.928 (0.895,0.962)	0.932 (0.901,0.965)	0.846∗
EGC	0.913 (0.870,0.956)	0.873 (0.755,0.990)	0.506∗
AGC	0.792 (0.737,0.847)	0.612 (0.454,0.771)	0.035∗

Note: LGIN: low-grade intraepithelial neoplasia; HGIN: high-grade intraepithelial neoplasia; EGC: early gastric cancer; AGC: advanced gastric cancer; CI: confidence interval; ∗Student *t* test was used.

## Data Availability

The data that support the findings of this study are available from the corresponding author upon reasonable request.
